# Supplementary material for: Antibiotic Use in Pediatrics: Perceptions and Practices of Romanian Physicians
Source: Antibiotics (Basel). 2025 Sep 27;14(10):976. doi: 10.3390/antibiotics14100976 (PMC12561463; doi:10.3390/antibiotics14100976)
Supplement: Supplementary file 1 [file antibiotics-14-00976-s001.zip › antibiotics-3859668-supplementary.pdf]

I. Demographic Data

This section gathered information on the physician's professional profile.

1. Specialty: Family physician / Pediatrician / General practitioner with pediatric competencies / Other specialty
2. Percentage of pediatric consultations: < 25% / 25–50% / 50–75% / > 75%
3. Primary practice environment: Urban / Rural / Mixed
4. Professional experience level: < 5 years / 5–15 years / > 15 years
5. Access to rapid diagnostic tests: Yes / No
6. Antibiotic stewardship course attendance: Have you attended an "antibiotic stewardship" course in the last three years? Yes / No

II. Practice and Perception of Antibiotic Therapy in Children

Respondents indicated their level of agreement on a 5-point Likert scale (1 – Totally disagree, 2 – Disagree, 3 – Neutral, 4 – Agree, 5 – Totally agree) with the following statements. While the questions in the original questionnaire were randomly shuffled to prevent influencing responses and to minimize the risk of "response bias," for a clear and logical presentation of the data, they are grouped here into the following thematic categories: Objective factors and Subjective factors.

A. Objective factors influencing antibiotic prescriptions

- Q1: The decision to prescribe antibiotics for children is primarily based on clinical symptoms.
- Q4: Fever above 38.5°C is often a sufficient reason for antibiotic therapy.
- Q5: Purulent secretions (nasal, bronchial, etc.) frequently lead to the initiation of antibiotic treatment.
- Q6: Acute otitis media justifies immediate initiation of antibiotic therapy in most cases.
- Q15: I request paraclinical tests (CRP, complete blood count) before prescribing antibiotics in uncertain cases.
- Q16: I use rapid tests (e.g., strep test, COVID19/Flu/RSV tests) in my current practice.
- Q17: Lack of access to rapid tests sometimes leads me to prescribe empirical antibiotics.
- Q18: I prefer to prescribe antibiotics when faced with an uncertain clinical picture.

B. Subjective factors influencing antibiotic prescriptions

- Q2: I primarily follow guidelines when deciding to initiate antibiotic treatment.
- Q3: Personal experience carries more weight than guidelines in uncertain cases.
- Q7: Parents frequently request antibiotics for common symptoms or viral infections.
- Q8: Parental pressure sometimes influences my prescribing decision.
- Q9: I try to explain to parents when antibiotics are not necessary.
- Q10: I sometimes prescribe antibiotics, with the instruction to administer them if symptoms worsen.
- Q11: Parents explicitly ask me to prescribe an antibiotic.
- Q12: Parents suggest that only antibiotics have worked for their child in the past.
- Q13: When parents express doubts that it is a viral infection, I am more inclined to prescribe an antibiotic.
- Q14: Parents' tone, language, or insistence sometimes leads me to prescribe an antibiotic even without a clear indication.
- Q19: I believe that in Romania, more antibiotics are prescribed to children than necessary.

- Q20: Factors like limited consultation time or lack of follow-up influence the prescribing decision.
- Q21: I frequently encounter cases where parents do not follow the prescribed antibiotic treatment.
- Q22: I believe that the duration of prescribed antibiotic treatment is often too long.
- Q23: I am interested in attending courses on rational antibiotic use.
